# Experimental dataset on the application of aloe vera gel as a natural coagulant for water treatment

**DOI:** 10.1016/j.dib.2025.112366

**Published:** 2025-12-09

**Authors:** Danieli Soares de Oliveira, Clainer Bravin Donadel

**Affiliations:** a Federal Institute of Espírito Santo - campus Cariacica, Rodovia Governador José Sette, 184, 29150-410, Cariacica, Espírito Santo, Brazil; b Federal Institute of Espírito Santo - campus Vitória, Avenida Vitória, 1.729, 29040-780, Vitória, Espírito Santo, Brazil

**Keywords:** Jar test experiments, Sustainable treatment technologies, Turbidity removal, Water clarification

## Abstract

This paper presents a comprehensive dataset from laboratory jar test experiments evaluating the performance of *Aloe vera* gel as a natural coagulant for water treatment, focusing on turbidity reduction. The coagulant was prepared through gel extraction, homogenization with tap water, and paper filtration. Synthetic turbid water was produced by dispersing bentonite in tap water to reach initial turbidity levels of 50, 100, 200, and 300 NTU. Coagulation tests were conducted under controlled laboratory conditions using a jar test apparatus, including rapid mixing, coagulant dosing, floc formation, and a 60-minute sedimentation stage. Turbidity was measured with a calibrated turbidimeter at 10-minute intervals for one hour, generating a dataset that records initial and residual turbidity values for different coagulant dosages. The dataset is organized in tabular format and includes metadata on coagulant preparation, synthetic water formulation, and operational parameters to ensure reproducibility. The results confirmed a consistent and significant reduction in turbidity across all tested conditions, demonstrating the effectiveness of *Aloe vera* gel as a natural coagulant. These data can support comparative studies on natural coagulants, modeling of coagulation dynamics, optimization of treatment conditions in low-resource contexts, and the validation of sustainable alternatives to chemical coagulants.

## Graphical abstract

**Figure:**
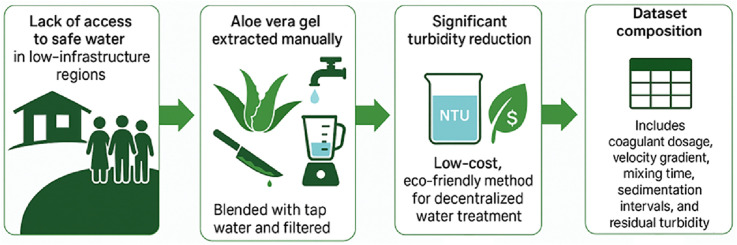

Specifications Table

**Table:** 

Subject	Earth & Environmental Sciences
Specific subject area	Water treatment using natural coagulants.
Type of data	Table (.xlsx format). Raw data.
Data collection	The dataset was generated under controlled laboratory conditions using a jar test apparatus (Athon, model JTAT6J2LDIG-CS), a turbidimeter (AKSO, model TU430), and a pH meter (Milwaukee Instruments, model MW150 MAX). *Aloe vera* gel was extracted, homogenized with tap water, filtered, and prepared as a liquid coagulant. Synthetic water was produced with bentonite suspensions adjusted to target turbidity levels. Residual turbidity was recorded at defined sedimentation times (0–60 min) and across different coagulant dosages, with all measurements performed in triplicate to ensure reproducibility.
Data source location	IFES - Federal Institute of Espírito Santo (Brazil).
Data accessibility	Repository name: Dataset on turbidity reduction using *Aloe vera* gel as a natural coagulant in water treatment Data identification number: 10.5281/zenodo.17041192 Direct URL to data: https://doi.org/10.5281/zenodo.17041192
Related research article	Oliveira, D.S.; Nascimento, R.S.; Donadel, C.B. Aloe Vera in Water Treatment: Toward a Greener Future for Environmental Engineering. Sustainability, 2025. 17, 4163. DOI: 10.3390/su17094163.

## 1 Value of the Data

• The dataset provides systematically collected turbidity measurements, including both initial and residual values, obtained under controlled jar test conditions using *Aloe vera* gel as a natural coagulant. These records offer raw, quantitative, and reproducible data documenting coagulant performance under varying operational conditions.
• The coagulant was prepared through a simple, low-cost method involving manual gel extraction, homogenization with tap water, and paper filtration, which makes the procedure easily replicable in different laboratory contexts.
• Researchers can reuse these data to quantitatively compare the efficiency of *Aloe vera* with other natural or chemical coagulants under similar laboratory setups, supporting benchmarking studies and cross-validation of coagulation performance.
• The dataset includes detailed metadata on coagulant preparation, synthetic turbid water formulation, dosage levels, and operational parameters (mixing speed, velocity gradient, and sedimentation time), facilitating full experimental replication and promoting methodological transparency.
• The turbidity data across different initial levels (50, 100, 200, and 300 NTU) enable the development and validation of computational models of coagulation and sedimentation, as well as optimization studies in water treatment simulations.
• The publication of these data will particularly benefit researchers, environmental engineers, and practitioners working in sustainable water treatment, as well as educators and students seeking accessible, well-documented datasets for training and methodological development, especially in contexts with limited infrastructure.

## 2 Background

Among the key stages of water treatment, coagulation plays a fundamental role in removing suspended solids and reducing turbidity, contributing to improved water quality. Traditional water treatment practices predominantly rely on chemical coagulants such as aluminum and iron salts. Although widely used for their effectiveness, these compounds may lead to secondary contamination and environmental impacts, including the accumulation of non-biodegradable sludge and potential health risks associated with residual metals in treated water [[1], [2], [3]].

In contrast, natural coagulants derived from plant-based materials have gained attention for offering a more sustainable and eco-friendly alternative. Several studies have highlighted the potential of different plant extracts as effective coagulants for the removal of turbidity and other contaminants, with *Aloe vera* standing out as a promising option [[4], [5], [6], [7], [8], [9]]. The coagulation mechanism of *Aloe vera* is mainly attributed to hydroxyl, carboxyl, and amide groups that promote charge neutralization and adsorption–bridging with negatively charged particles, enhancing floc formation [5]. Reported turbidity removal efficiencies reach up to 88 %, comparable to Moringa oleifera and Cactus opuntia (80–95 %). Moreover, *Aloe vera* is widely available, inexpensive, biodegradable, and easily extracted using water as a solvent, making it suitable for decentralized and low-cost water treatment applications, particularly in developing regions.

## 3 Data Description

The dataset compiles systematic laboratory measurements from jar tests designed to evaluate *Aloe vera* gel as a natural coagulant for water treatment. The data capture variations in coagulant dosage, coagulation/flocculation time, mixing speed, global velocity gradient, sedimentation time, and replicate turbidity measurements. Two mixing speeds (120 and 151 rpm) and their corresponding global velocity gradients (176 and 248 s⁻¹) were intentionally selected to represent different hydraulic energy inputs during the coagulation/flocculation stage, following standard jar test procedures. These values reflect the operational characteristics of the real coagulation/flocculation unit available at the research laboratory where the study was conducted. These variations were deliberately introduced as controlled experimental factors to assess the influence of different hydraulic conditions, and were not caused by equipment-related limitations. Coagulant dosage, sedimentation time, and hydraulic conditions (mixing speed and velocity gradient) were the systematically varied experimental parameters, whereas temperature and sample volume were kept constant throughout all trials. The dataset follows a structured tabular format, where each row represents a single experimental observation. Table 1 describes the variables included in the dataset, their units, and the type of information they provide. This structure ensures reproducibility and facilitates statistical analysis, modeling, and comparison across different operational conditions. To illustrate the dataset structure, examples are provided in Table 2. These examples highlight how the information is organized, while the complete dataset offers full coverage of all trials and replicates.

**Table 1:** Variables and descriptions of the turbidity dataset.

Variable	Unit	Description
Coagulant dosage	mL/L	Volume of *Aloe vera* gel applied in each jar during the experiment. Values range from 10 to 120 mL/L, with each jar corresponding to a distinct dosage.
Coagulation/flocculation time	minutes	Duration of the rapid mixing stage prior to sedimentation. Recorded values are typically 1.0 or 1.11 min depending on the setup.
Mixing speed	rpm	Mixing speed used during the coagulation/flocculation stage. Reported values include 120 rpm and 151 rpm.
Global velocity gradient	s^-1^	Global velocity gradient applied during the coagulation/flocculation stage. Reported values include 176 s^-1^ and 248 s^-1^.
Trial number	–	Identifier of the experimental replicate. Each condition was tested in triplicate, labeled as 1, 2, or 3.
Sedimentation time	minutes	Time elapsed during quiescent settling before turbidity measurement. Values include 0 (initial turbidity), 10, 20, 30, 40, 50, and 60 min.
Turbidity	NTU	Measured residual turbidity of the water sample after sedimentation. Expressed in Nephelometric Turbidity Units (NTU).

**Table 2:** Example of residual turbidity data at different initial turbidity levels and coagulant dosages.

Coagulant dosage [mL/L]	Coagulation/flocculation time [min]	Mixing speed [rpm]	Global velocity gradient [s^-1^]	Trial number [-]	Sedimentation time [min]	Turbidity [NTU]
30	1	120	176	1	0	58.5
35	1	120	176	1	0	52.2
40	1	120	176	1	0	53.4
45	1	120	176	1	0	58.4
50	1	120	176	1	0	52.2
55	1	120	176	1	0	55
…	…	…	…	…	…	…
30	1	120	176	1	10	46.3
35	1	120	176	1	10	47.6
40	1	120	176	1	10	44.9
45	1	120	176	1	10	45.5
50	1	120	176	1	10	41.8
55	1	120	176	1	10	37.1
…	…	…	…	…	…	…
30	1	120	176	1	20	32.5
35	1	120	176	1	20	25.2
40	1	120	176	1	20	22.4
45	1	120	176	1	20	23.1
50	1	120	176	1	20	19.77
55	1	120	176	1	20	20
…	…	…	…	…	…	…
30	1	120	176	1	30	23.5
35	1	120	176	1	30	20.4
40	1	120	176	1	30	20.7
45	1	120	176	1	30	20.7
50	1	120	176	1	30	20
55	1	120	176	1	30	16.59
…	…	…	…	…	…	…
30	1	120	176	1	40	18.35
35	1	120	176	1	40	15.62
40	1	120	176	1	40	14.65
45	1	120	176	1	40	18.01
50	1	120	176	1	40	15.11
55	1	120	176	1	40	12.5
…	…	…	…	…	…	…
30	1	120	176	1	50	16.42
35	1	120	176	1	50	10.85
40	1	120	176	1	50	10.62
45	1	120	176	1	50	12.1
50	1	120	176	1	50	9.6
55	1	120	176	1	50	8.29
…	…	…	…	…	…	…
30	1	120	176	1	60	13.92
35	1	120	176	1	60	10.79
40	1	120	176	1	60	9.88
45	1	120	176	1	60	7.67
50	1	120	176	1	60	9.65
55	1	120	176	1	60	9.94
…	…	…	…	…	…	…

## 4 Experimental Design, Materials and Methods

This section presents a detailed description of the dataset generated from laboratory experiments evaluating the application of *Aloe vera* gel as a natural coagulant for water treatment. To validate the alternative method for producing the *Aloe vera*-based natural coagulant, turbidity removal efficiency was used as the primary performance indicator. Turbidity is a key parameter in assessing the effectiveness of coagulation, flocculation, and sedimentation processes in water treatment systems. The dataset was designed to provide systematically collected turbidity values under controlled jar test conditions, using synthetic water samples with initial turbidity levels of 50, 100, 200, and 300 NTU. Residual turbidity was measured at defined sedimentation times to capture the performance of the coagulant across different dosages.

**Fig. 1:**
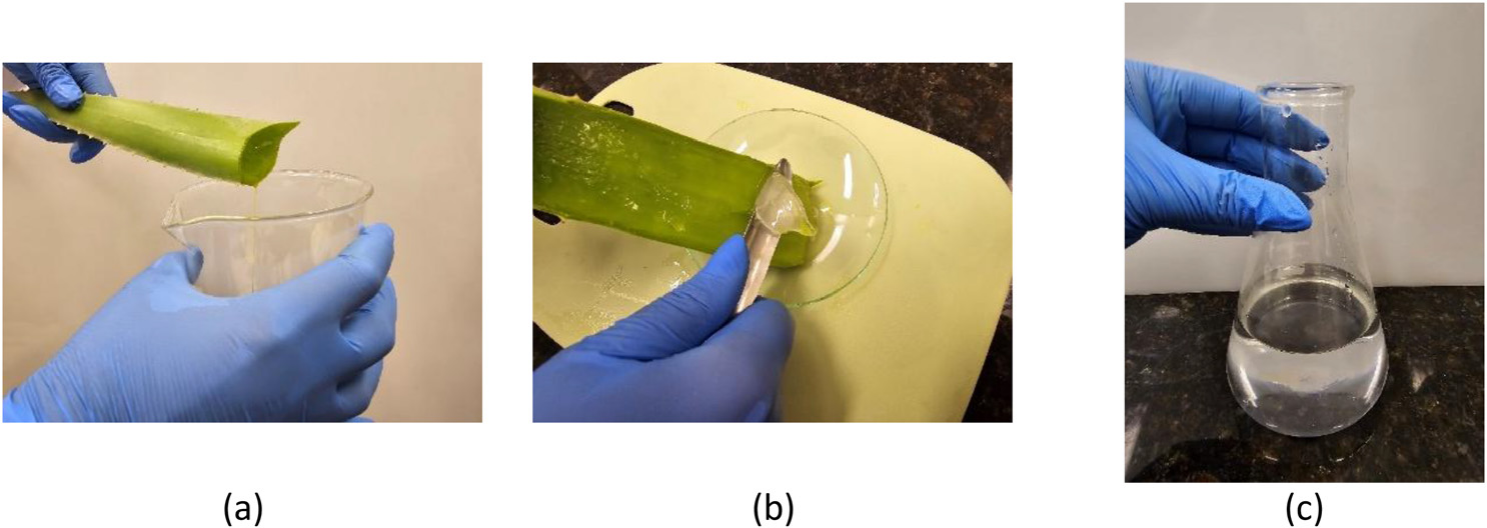
Main steps in the preparation of the Aloe vera-based natural coagulant: (a) draining of aloin after leaf cutting, (b) removal of the outer rind and extraction of the inner gel, and (c) final liquid coagulant used in coagulation tests.

The preparation of the coagulant began with the selection of a healthy, mature *Aloe vera* leaf, which was manually removed from the base of the plant using a clean, sharp knife. The leaf was then placed at an angle over a beaker to allow the yellow liquid (aloin) to drain, as it is not used in the coagulation process - Fig. 1a. The outer rind was carefully removed with a longitudinal cut to expose the inner gel, which was extracted with a spatula by gently sliding it along the inner surface of the opened leaf - Fig. 1b The extracted gel was transferred to a pre-tared watch glass and weighed on an analytical balance to quantify the amount used in the coagulant preparation. The measured gel was then placed in a blender, and 2 g of *Aloe vera* gel were diluted in 50 mL of tap water, resulting in a final concentration of 40 mg/mL. The mixture was homogenized at approximately 25 °C (room temperature) and pH ≈ 7 for about 30 s at medium speed. Following homogenization, the solution was subjected to simple filtration using filter paper (average pore size of approximately 10 µm) to remove residual solid particles, resulting in the liquid coagulant, which was transferred to a clean, properly labeled container for subsequent use in coagulation tests - Fig. 1c.

Synthetic water was prepared to simulate turbid conditions under controlled laboratory settings. Bentonite, a clay commonly used for its stability and ability to form colloidal suspensions, was employed as the turbidity-inducing agent. Natural sodium bentonite supplied by INDUPROPIL (Brazil) was used due to its high swelling capacity, fine particle size distribution, and ability to produce stable and reproducible turbidity levels, making it suitable for coagulation and flocculation studies. Specific amounts of bentonite were dispersed in 2 L of tap water, with concentrations adjusted to achieve target turbidity levels. The suspensions were stirred for 30 min to ensure homogeneity and then stored for at least 24 h to allow particle stabilization. Before each jar test, turbidity was measured to confirm that variations over the 24-hour period remained within ±5 % of the initial value, ensuring stability and consistency of the synthetic turbid water used in all experiments.

**Fig. 2:**
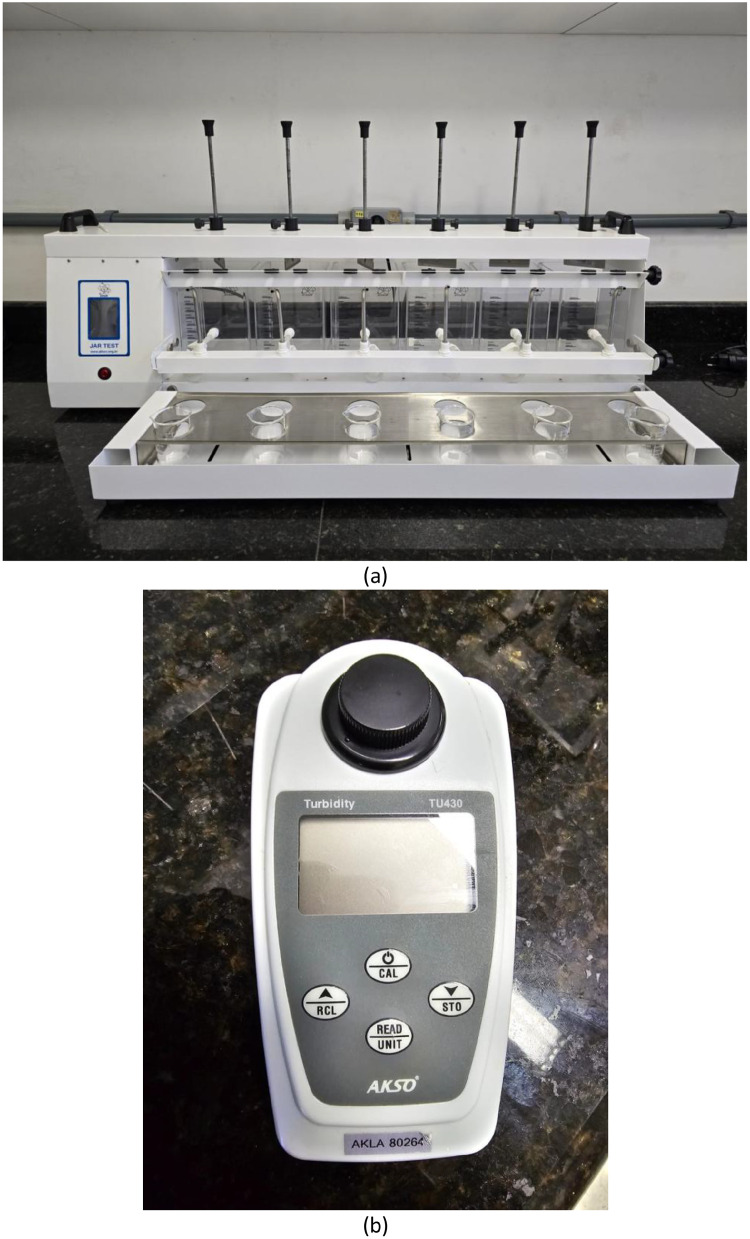
Experimental setup: (a) jar test apparatus and (b) turbidimeter used for turbidity measurements.

Jar test experiments were conducted under controlled laboratory conditions to assess turbidity removal using the *Aloe vera*-based coagulant. A jar test apparatus with six 2 L beakers was filled with synthetic water (Fig. 2a). The operational parameters of the coagulation test—rapid mixing at 151 rpm for 1.11 min—were adopted in accordance with the procedure described in [7]. The procedure included rapid mixing to promote coagulation and floc formation, followed by a 60 min sedimentation period. Turbidity was measured at 10 min intervals with a calibrated turbidimeter (Fig. 2b), and all tests were performed in triplicate to ensure reproducibility. The resulting dataset provides residual turbidity values across different dosages and initial turbidity conditions.

## Limitations

Some limitations of this dataset should be acknowledged. The turbidity data were obtained exclusively under laboratory conditions using synthetic water prepared with bentonite, which does not capture the full variability and complexity of natural water matrices. The dataset is therefore limited to controlled conditions and may not reflect variations caused by organic matter, pathogens, or heavy metals commonly present in real water sources.

The dataset also focuses solely on turbidity as the measured parameter, without including additional indicators of water quality such as pH, conductivity, or microbiological parameters. Furthermore, the data represent short-term laboratory experiments, and do not include information on the long-term stability of the *Aloe vera*-based coagulant or its behavior in larger-scale operations.

These limitations should be considered when reusing the dataset, as the values are specific to the described experimental conditions and parameters.

## Ethics Statement

The authors confirm that they have read and comply with the ethical requirements for publication in Data in Brief, and that the current work does not involve human subjects, animal experiments, or any data collected from social media platforms.

## Credit Author Statement

**Danieli Soares de Olivera:** Conceptualization, Methodology, Validation, Formal analysis, Investigation, Resources, Data Curation, Writing - Original Draft, Writing - Review & Editing, Visualization, Supervision, Project administration, Funding acquisition. **Clainer Bravin Donadel:** Software, Formal analysis, Data Curation, Writing - Original Draft, Writing - Review & Editing.

## Data Availability

ZenodoDataset on turbidity reduction using Aloe vera gel as a natural coagulant in water treatment (Original data).
